# Supplementary material for: Learning curve and surgical outcome of robotic assisted colorectal surgery with ERAS program
Source: Sci Rep. 2022 Nov 29;12:20566. doi: 10.1038/s41598-022-24665-w (PMC9709162; doi:10.1038/s41598-022-24665-w)
Supplement: Supplementary file 1 — Supplementary Information 1. [file 41598_2022_24665_MOESM1_ESM.zip › LSC Raw data-Ñ[▒K/069/H_ñw╝╨░O▒Kñσ.pdf]

正式摘要(電子簽章：000000186481102)	【讀取】憑證	
W95 / W95 ☐ 英文標題(注意:無英文姓名-請補病人基本檔!)	☐	

臺中榮民總醫院
出院病歷摘要

轉入號 : 轉出號 :

[redacted]

(8)流水編號	
(9)入院日期	109/02/18 (2020) 直腸外科 W95 - 39
(10)轉科日期	
(11)出院日期	109/02/27 (2020) 住院天數計 9 日

診斷

- 入院 :Sigmoid colon, Adenocarcinoma, moderately differentiated
  PH:
  1.Hypertension under Norvasc 1# QD
  2.Hyperlipidemia under Lipitor 1# QD
  3.Osteoporosis
  4.acute L1 compression fracture with kyphosis s/p Percutaneous vertebroplasty on 2015/6/19
  5.T11 compression fracture, caused by drop attack in 2010 s/p REH
  6.s/p appendectomy 10 years ago.
- 出院 :. Sigmoid colon, pT3N1, Adenocarcinoma, moderately differentiated
  s/p Laparoscopic Anterior resection and anastomosis on 2020/2/19
  PH:
  1.Hypertension under Norvasc 1# QD
  2.Hyperlipidemia under Lipitor 1# QD
  3.Osteoporosis
  4.acute L1 compression fracture with kyphosis s/p Percutaneous vertebroplasty on 2015/6/19
  5.T11 compression fracture, caused by drop attack in 2010 s/p REH
  6.s/p appendectomy 10 years ago.

主訴

- streaks of blood on the tissue after wiping since 2020/1

病史

- This 72-year-old woman had hx of
  1.Hypertension under Norvasc 1# QD
  2.Hyperlipidemia under Lipitor 1# QD
  3.Osteoporosis
  4.acute L1 compression fracture with kyphosis s/p Percutaneous vertebroplasty on 2015/6/19
  5.T11 compression fracture, caused by drop attack in 2010 s/p REH
  6.s/p appendectomy 10 years ago.
  Her ADL was totally independent. This time, streaks of blood on the tissue after wiping on 2020/1 at home. She had history of internal hemorroid but no bleeding before. Bowel habit change was for one year. (stool became thiner but no diarreha). She denied abdominal pain, diarrhea, constipation, dizziness, dyspnea, body weight change recently. Due to streaks of blood on the tissue, she went to our CRS OPD for help on 2020/1/20. Therefore, abdominal CT showed segmental wall thickening of the sigmoid colon and two low attenuation nodules in bilateral lobes of the liver and suggested MRI of the liver for further evaluation. Colonosocpy showed ""colonoscopy was up to T-colon,1.colitis 30 cm FAV, s/p biopsy.2.tumor 25 cm FAV, s/p biopsy."" Pathology showed Adenocarcinoma, moderately differentiated. Pend Liver MRI formal report but favor liver cyst now. PET CT was also peroformed showed "Compatible with cancer of sigmoid colon " Therefore, under the impression of sigmoid colon cancer, she was admitted for Lapa AR on 2020/2/19.

體檢發現

- Anal inspection: int. hemorhoid.
  cos: tumor 25 cm FAV, colitis 30 cm FAV.
  CEA: 5.9

-
  - General appearance: On examination, the patient was a well nourished , female , with fair -looking, , consciousness : clear , alert
  - Statistics and Vital signs:
date	體溫(BT)	脈搏(P)	呼吸(R)	血壓(BP)	身高(BH)	體重(BW)	BMI
20200218	37℃	57次/分	19次/分	139/63mmHg	158.1cm	74.4kg	29.8
    - body weight : 60 kg
    - height : 156 cm
    - B.P. 139/63 mmHg , P.R. 57 /min , R.R. 19 /min , B.T. 37 ℃
  - Skin: Normal skin color & appearance: - , no skin rash , no petechia , no ecchymosis
  - HEENT: Pupils size : 3 / 3 mm , light reflex : + / + , no facial asymmetry , no nasal or ear discharge , no Neck stiffness , no lymphadenopathy , no jugular vein engorgement
  - Chest :
    - Inspection : no accessory muscle respiration , no paradoxical movement
      no wall deformity , normal respiratory pattern , no gynecomastia , no increased A-P diameter , no operation scar , no narrow or widening of intercostal space , no spider angiomata
    - Palpation : no tender point , no palpable mass , no abnormal vocal fremitus
      no subcutaneous emphysema , no subcutaneous crepitance , no breast nodule , no axillary lymphadenopathy
    - Percussion : no abnormal dullness , no hyper-resonance
    - Auscultation : no rales , no rhonchi , no wheezing
      clear breathing sound , no peripheral bronchial sound , no friction rub
  - Heart :
    - Inspection : no visible apical impulse
    - Palpation : no thrill , no heave
      palpable apical impulse at 5th ICS at left mid-clavicular line.
    - Percussion : dullness between 2nd to 5th ICS.
    - Auscultation : no irregular heart beats , no ectopic beats , no S3 , no S4 , no murmur , no gallop rhythm
      no pericardial friction rub , no opening snap , no click
  - Abdomen :
    - Inspection : no distension , no Cullen's sign , no Turner's sign , no spider angiomata , no superficial vein engorgement
      flat , no ecchtmosis , no patechiae , no striae , no caput medusae , no operation scar , no hernia
    - Auscultation : Normoactive bowel sound
      no bruit , no succusion splash sound , no metallic sound
    - Percussion : no shifting dullness , no fluid wave , no central tympanic , no knocking pain
      liver span: -
    - Palpation : no tenderness , no muscle guarding , no rebounding pain , no palpable abdominal mass , no hepatomegaly , no splenomegaly , no Murphy's sign
      soft , no hernia , no bladder distension , no inguinal lymphadenopathy
  - Back and Extremities: no Clubbed fingers , no pitting edema , no deformity , no spontaneous movement
  - Exogenitalia and anus: normal

手術日期及方法

- 手術日期及方法20200219 Laparoscopic Anterior resection and anastomosis - malignant

住院治療經過

- After admission, Laparoscopic Anterior resection and anastomosis was performed smoothly on 2020/2/19. Procedure was performed smoothly without complication. She can do off-bed acitivity with mild wound pain (NRS:3) since post-opeartive day 1. NG was removed on 2/20 and Foley was removed on 2/21 smoothly. We let her try liquied diet on 2/23 and semi-liquid diet on 2/24. Because mild abdominal pain and KUB showed "subphrenic air which might be post-operative related and bowel distension" No peritoneal sign and her drain didn't found stool-like material. Stool passage 3 times on 2/24 night and abdominal pain improved on 2/25 morning. Drain color showed mild turbid with yellow-green color on 2/25 but patient denied any discomfort. Lab data on 2/26 showed no leukocytosis. Therefore, we shifted to soft diet on 2/26 and removed her drain and stiches. Due to stable condition, she was discharged today and arranged OPD follow-up on 3/2.

合併症

- nil

一般檢查

- 累積報告：

DATE	TIME	ALB	TP	BIL.T	BIL.D	ALKP	AST	ALT	LDH	CHOL	TG	HDL-C	LDL	UA	NH3	AMY
1090219	1335	3.6		0.65	0.12		27	13								
1090224	0931	3.5		0.6		61	20	9								
1090226	0857			0.5		67	21	8								

DATE	TIME	WBC	RBC	HGB	HCT	MCV	PLT	BLST	NEUT	BAND	SEG	LYM	MON	EOS	BAS
1090219	1335	9720	3.66	10.7	31.9		213		92.8			4.6	2.5	0.0	0.1
1090224	0925	3750	3.63	11.0	31.7	87.3	259		70.3			18.7	7.5	3.2	0.3
1090226	0857	4300	3.83	11.2	33.3	86.9	286		72.2			16.7	7.9	3.0	0.2

DATE	TIME	NA	K	CL	CA	BUN	CREAT	Mg	P	CRP	ESR	PCT	LIPASE	LACTATE
1090219	1335	132	3.6			6	0.50							
1090224	0931	137	3.7	101	8.1	10	0.59	1.6		2.748				
1090226	0819													6.7
1090226	0857	136	3.7	100	8.2	9	0.64	1.6						

DATE	TIME	PH	BEB	SCVO2	PO2	HCO3	HGB	PCO2	TCO2
1090224	1009	7.402	0.4	74.0	39.2	25.2		41.4	26.5
1090226	0820	7.391	2.6	31.6	20.2	28.1		47.3	29.6

- 放射線報告：檢驗項目 **:CHEST PA VIEW** 報告日期 **:1090225**

  主　訴：
  Result:
  Finding:
  >> Pneumoperitoneum, Suggest clinical correlation.
  > Enlarged cardiac shadow
  > Tortuosity and atherosclerotic changes of T-aorta
  > Right CP angle blunting, pleural effusion or pleural change could be considered.
  > Prominence of bilateral hila with increased lung markings over bilateral lower lung fields
  > Degenerative change of thoracic and lumbar spine with osteophytes formation.
  > S/P cemented vertebroplasty of L1.
  > Generalized osteoporosis.
  Impression:
  As above description.

特殊檢查

病理報告

- 活體組織切片病理報告　手術日期：**109/02/19**　病理號：**S0905351**

診斷醫師:文美卿 報告日期:109/02/26
Pathologic diagnosis:
1. Sigmoid colon, laparoscopic anterior resection ---
a. Adenocarcinoma, moderately differentiated. Tumor invaded to mesocolic soft tissue and metastasized to mesocolic lymph nodes (1/31). The bilateral cut ends are free of tumor.
b. Diverticulum.
2. IMA root, dissection --- Unremarkable fibroadipose tissue.
3. Colon, cut end, distal, resection --- Unremarkable.
Ancillary study for diagnosis:
IHC stains for MLH1, MSH2, PMS2 and MSH6 are done on section H. All above markers are preserved.
Prognostic and predictive factor:
1. Tumor size: 5x4 cm.
2. Depth of invasion: Mesocolic soft tissue
3. Histologic grade: G2: Moderately differentiated.
4. Angiolymphatic invasion: Not identified.
5. Perineural invasion: Not identified.
6. Tumor deposits (discontinuous extramural extension): Not identified.
7. Macroscopic tumor perforation: Not identified.
8. Surgical Margins: a) Proximal Margin: Uninvolved by invasive carcinoma, distance of tumor from margin: 8 cm. b) Distal Margin: Uninvolved by invasive carcinoma, distance of tumor from margin: 5 cm. c) Radial or Mesenteric Margin: Uninvolved by invasive carcinoma, distance of tumor from margin: 5 cm.
9. Lymph node status: Involved (1/31).

- 10. Extranodal involvement: Not identified.
  11. Tumor regression grading S/P CCRT: No known presurgical therapy.
  12. Type of polyp in which invasive carcinoma arose: Not identified.
  13. Additional pathologic findings: Not identified.
  14. Pathological TNM Stage: pT3N1(According to the eighth edition, American Joint Committee on Cancer Staging Guidelines for Tumors).
  15. TNM descriptors: Not applicable.
  Gross description:
  The specimen consists of 1) a segment of colon, 17 cm in length and 6 cm in proximal cut end circumference, 6 cm in distal circumference, and mesocolic soft tissue. A gray tan firm annular elevated and centrally ulcerative tumor, 5x4 cm, is noted, 5 cm to the distal cut end. On cross section, the tumor invades to the mesocolic soft tissue with 5 cm to the radial margin. The both cut ends are free of tumor grossly. 2) IMA root, a piece of tan yellow soft tissue, 6x4x2 cm. 3) distal cut end, a piece of tan mucosa-covered soft tissue, 3x2x1 cm. Representative part for section: A-B) specimen 2 C) specimen 3 D) proximal cut end E) radial margin F-K) tumor L-N) lymph nodes.
  Note:
  1. The interpretation of MMR panel and recommended studies are as below: (1) No loss of nuclear expression of MMR proteins: low probability of microsatellite instability-high (MSI-H) . (2) Please note: There are exceptions to the above IHC interpretations. These results should not be considered in isolation, and clinical correlation with genetic counseling is recommended to assess the need for germline testing.
  2. This case has been peer reviewed by two doctors.
  #T-59470_2 #M-81403_2 2 1105 S0000F
  #T-59470_2 #M-32700_2 0 1105 000000
  #T-C4560_2 #M-81406_2 9 1105 000000

其它

出院時情況

- 3.改門診治療

出院指示

藥名	劑量	劑量單位	用法	頻次	數量	數量單位	現況	天數	單位
Smecta powder 3g	1	PKG	PO	TIDPRN	15	PKG	TH	5	天
$Miyarisan BM powd.	1	PACK	PO	TID	42	PACK	TH	14	天

出院計劃

主治醫師 2222H 林俊余 / 住院醫師 1741F 侯順方

正式摘要(**電子簽章：000000186481102)**

W95 / W95

doctorRank:B
